# 1806. Community Spread of Highly Resistant Enterobacterales

**DOI:** 10.1093/ofid/ofad500.1635

**Published:** 2023-11-27

**Authors:** Jamie Xiao, Dylan Brown, Liang Chen, Thomas Holowka, Laura Ruegsegger, Mera F Liccione, Robert A Bonomo, Steven Marshall, Susan D Rudin, Luther A Bartelt, David van Duin

**Affiliations:** University of North Carolina, Chapel Hill, North Carolina; University of North Carolina at Chapel Hill, Chapel Hill, North Carolina; HMH-CDI, Nutley, New Jersey; University of North Carolina, Chapel Hill, Chapel Hill, North Carolina; University of North Carolina at Chapel Hill, Chapel Hill, North Carolina; University of North Carolina at Chapel Hill, Chapel Hill, North Carolina; Case Western Reserve University, Cleveland, Ohio; Louis Stokes Cleveland VA, Cleveland, Ohio; Case Western Reserve University, Cleveland, Ohio; University of North Carolina School of Medicine, Chapel Hill, NC; University of North Carolina at Chapel Hill, Chapel Hill, North Carolina

## Abstract

**Background:**

Highly Resistant (including ceftriaxone-resistant and carbapenem-resistant) Enterobacterales (HRE) are a major and growing public health threat. Community spread of HRE is poorly understood.

**Methods:**

Monthly stool samples were obtained from patients who were discharged home after a hospitalization at one of 6 participating US medical centers with a clinical culture positive for HRE based on local laboratory antimicrobial susceptibility testing. Stool samples were also collected from community and household contacts they referred, and any contacts referred by those contacts. Samples were screened by culture on selective media for HRE, and with PCR for presence of extended-spectrum β-lactamase (ESBL), AmpC, and carbapenemase genes. Whole genome sequencing was performed on selected isolates.

**Results:**

Of 1,923 patients with positive HRE cultures from healthcare settings, 943/1,923 (49%) were discharged home. 65/943 (7%) were consented into the study, and 31/65 (48%) returned at least one sample (median of 6 samples; IQR 5-10). The index HRE species was isolated from stool samples from 19/31 (61%) participants; the median duration of detected carriage was 391 days (range 84-662 days). Screening for resistance genes was performed on 477 sample-derived isolates (Table 1); ESBL/AmpC and carbapenemase genes were present in 221/447 (49%) and 17/447 (4%), respectively. Stool samples (median of 7 samples; IQR 5-11) were collected from 11 contacts of 9 participants, and 1 contact of a contact. Index HRE were isolated from samples from 10/12 contacts (83%); the median duration from date of positive index culture to last positive sample in a contact was 400 days (range 146-588). Whole genome analysis confirmed genetic similarity (< 21 single nucleotide polymorphism difference) between ceftriaxone-resistant *bla*_SHV_ and *bla*_CTX-M-15_ carrying *K. pneumoniae* isolates in a cluster of 4 participants, which included secondary transmission between a contact of the index patient and one of their contacts over a period of 555 days.

**Table.**
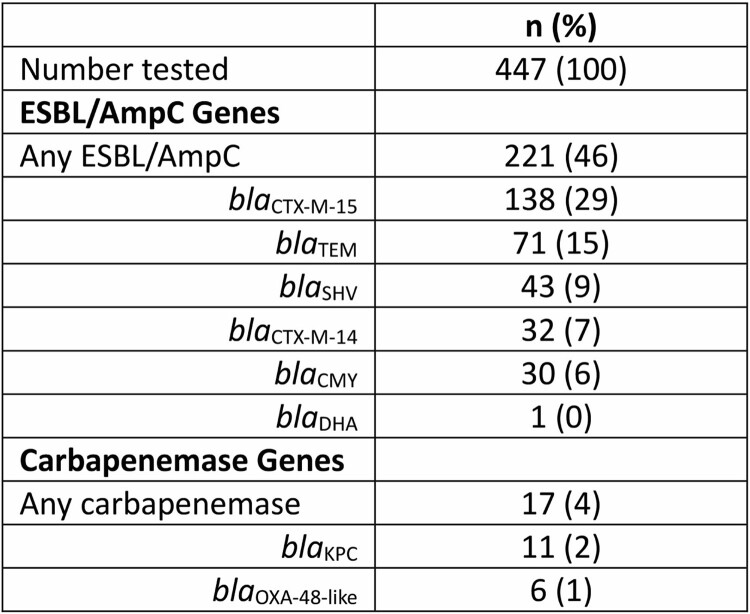
Resistance genes

**Conclusion:**

Patients with a clinical culture positive for HRE are at risk for prolonged intestinal HRE carriage after hospitalization. Upon returning home, community spread of HRE was very common in this cohort.

**Disclosures:**

**Robert A. Bonomo, MD**, Entasis: Grant/Research Support|Merck: Grant/Research Support|venatorax: Grant/Research Support|Wockhardt: Grant/Research Support **David van Duin, MD, PhD**, Entasis: Advisor/Consultant|Merck: Advisor/Consultant|Merck: Grant/Research Support|Pfizer: Advisor/Consultant|Pfizer: Honoraria|Qpex: Advisor/Consultant|Roche: Advisor/Consultant|Shionogi: Advisor/Consultant|Shionogi: Grant/Research Support|Union: Advisor/Consultant|Utility: Advisor/Consultant

